# Distinct clinical features between acute and chronic progressive parenchymal neuro-Behçet disease: meta-analysis

**DOI:** 10.1038/s41598-017-09938-z

**Published:** 2017-08-31

**Authors:** Mizuho Ishido, Nobuyuki Horita, Masaki Takeuchi, Etsuko Shibuya, Takahiro Yamane, Tatsukata Kawagoe, Takehito Ishido, Kaoru Minegishi, Ryusuke Yoshimi, Yohei Kirino, Shunsei Hirohata, Yoshiaki Ishigatsubo, Mitsuhiro Takeno, Takeshi Kaneko, Nobuhisa Mizuki

**Affiliations:** 1 Department of Ophthalmology and Visual Science, Yokohama City University Graduate School of Medicine, Yokohama, Japan; 2 Department of Pulmonology, Yokohama City University Graduate School of Medicine, Yokohama, Japan; 3 Department of Stem Cell and Immune Regulation, Yokohama City University Graduate School of Medicine, Yokohama, Japan; 4 Department of Rheumatology and Infectious Diseases, Kitasato University School of Medicine, Kanagawa, Japan; 5 Yokohama City University Graduate School of Medicine, Yokohama, Japan; 6 Department of Allergy and Rheumatology, Nippon Medical School Graduate School of Medicine, Tokyo, Japan

## Abstract

Neuro-Behçet’s disease (NBD) is subcategorized into parenchymal-NBD (P-NBD) and non-parenchymal-NBD types. Recently, P-NBD has been further subdivided into acute P-NBD (A-P-NBD) and chronic progressive P-NBD (CP-P-NBD). Although an increasing number of studies have reported the various clinical features of A-P-NBD and CP-P-NBD over the last two decades, there was a considerable inconsistency. Two investigators systematically searched four electrical databases to detect studies that provided sufficient data to assess the specific characteristics of A-P-NBD and CP-P-NBD. All meta-analysis was carried out by employing the random-model generic inverse variance method. We included 11 reports consisted of 184 A-P-NBD patients and 114 CP-P-NBD patients. While fever (42% for A-P-NBD, 5% for CP-P-NBD, p < 0.001, I^2^ = 93%) was more frequently observed in A-P-NBD cases; sphincter disturbances (9%, 34%, P = 0.005, I^2^ = 87%), ataxia (16%, 57%, P < 0.001, I^2^ = 92%), dementia (7%, 61%, P < 0.001, I^2^ = 97%), confusion (5%, 18%, P = 0.04, I^2^ = 76%), brain stem atrophy on MRI (4%, 75%, P < 0.001, I^2^ = 98%), and abnormal MRI findings in cerebellum (7%, 54%, P = 0.02, I^2^ = 81%) were more common in CP-P-NBD. Cerebrospinal fluid cell count (94/mm^3^, 11/mm^3^, P = 0.009, I^2^ = 85%) was higher in A-P-NBD cases. We demonstrated that A-P-NBD and CP-P-NBD had clearly different clinical features and believe that these data will help future studies investigating P-NBD.

## Introduction

Behçet’s disease (BD) is a multisystem inflammatory disease with unknown etiology. The classical frequent symptoms are uveitis, genital ulcers, skin lesions, and recurrent oral aphthous ulcers^1^. The prevalence of BD is high in the Middle East, the Mediterranean basin, and the Far East regions, but it is rare in northern Europe, the American continents, and southern Africa. Central nervous system (CNS) involvement in BD has remained one of the most serious complications of the disease since the first report by Knapp in1942^2^. The conception of NBD (neuro- Behçet’s disease) was proposed by Cavara and D’Ermo^3^. The frequency of NBD greatly varies from 1.3%^4^ to 59%^5^ depending on the reports. The general consensus is that approximately 10% of BD patients have neurological involvement^6, 7^.

In our current understanding, there are two clinical categories of NBD, parenchymal-NBD (P-NBD) and nonparenchymal-NBD (NP-NBD)^7–9^. P-NBD, which is caused by parenchymal pathology, accounts for the majority of NBD. Some experts call P-NBD as intra-axial NBD, primary-NBD, or simply “NBD”. On the other hand, NP-NBD, which is usually caused by occlusion or hemorrhage of the main vascular structures, or aneurysm in the CNS, is relatively rare. To indicate NP-NBD, some researchers use alternative wordings such as vasculo-NBD, secondary NBD, or extra-axial NBD. The frequency of this subtype is reported 10 to 20% of all NBD patients from UK^10^ and Turkey^11, 12^, whereas it was extremely rare in Japan^13, 14^. Parenchymal-NBD is featured by diffuse brainstem, cerebral, optic, and spinal cord symptoms. Abnormal sign can appear depending on the site of involvement, typically brainstem atrophy and cerebral abnormal signs are observed. On the other hand, cerebral venous thrombosis, pseudo-tumor like intracranial hypertension, and acute meningeal syndrome are common forms of nonparenchymal-NBD. Cerebral sinus or vein thrombosis and meningeal enhancement may be revealed for MRI imaging of nonparenchymal-NBD^7–9^.

Because P-NBD shows heterogeneous clinical pictures, which require different therapeutic strategies, several lines of clinical subtyping of P-NBD patients had been shown in various studies^7, 9, 11^. Hirohata *et al*. have proposed clinical-orientated and simple classification in which P-NBD is further classified into an acute type and chronic progressive type, depending on its clinical course^13, 15, 16^. Acute P-NBD (A-P-NBD) typically features acute and transient symptoms such as fever and hemiparesis accompanied by inflammatory features including elevated cell count in the cerebrospinal fluid (CSF), while chronic progressive P-NBD (CP-P-NBD) is characterized by ataxia, dementia, incontinence, and brainstem atrophy^17, 18^. It is sometimes difficult to clearly classify some P-NBD cases into A-P-NBD and CP-P-NBD categories. A portion of P-NBD patients can experience acute first attack and following chronic progressive course. Over the last two decades, an increasing number of studies have reported the various clinical features of A-P-NBD and CP-P-NBD. However, these reports indicated inconsistencies in the prevalence of symptoms, magnetic resonance imaging (MRI) findings, and laboratory results. Therefore, we designed this systematic review and meta-analysis to reveal the key features of A-P-NBD and CP-P-NBD and to clearly differentiate them.

## Methods

### Study overview

This study was conducted following the standard method of meta-analysis^19^. Institutional review board approval and patient consent were not required because of the review nature of this study.

### Eligibility criteria

We planned to include case-series, cohort studies, case-control studies, cross-sectional studies, and randomized trials that provided sufficient data to assess the specific characteristics of A-P-NBD and CP-P-NBD. To calculate the pooled value, each study had to include at least two cases in a disease category. Thus, a single-case report was excluded. The reports had to be published as English full articles. Non-English reports and conference abstracts were excluded. Review articles without original data were also excluded. A study should assess at least one of the demographic characteristics, symptoms, MRI findings, and laboratory data listed in the Table 1. Reports that described P-NBD patients with a specific co-morbidity or a symptom were excluded. For example, a study that included only patients who had both A-P-NBD and headache was excluded. A study that evaluated P-NBD and NP-NBD collectively was not accepted. Although diagnostic criteria proposed by the International Study Group for Behcet’s Disease 1990 and that by International consensus 2014 was preferred^7, 20^, other criteria were also accepted.

**Table 1:** Characteristics of included studies.

Author	Year	Country	BD definition	A-P-NBD	CP-P-NBD
Akman-Demir^23^	2008	Turkey	ISGBD criteria 1990	26	14
Coban^24^	1999	Turkey	ISGBD criteria 1990	12	0
De Cata^25^	2007	Italy	ISGBD criteria 1990	0	2
Haghighi^45^	2011	Iran	ISGBD criteria 1990	0	8
Hirohata^14^	2012	Japan	ISGBD criteria 1990	76	35
Kanoto^27^	2013	Japan	ISGBD criteria 1990	0	4
Matsui^28^	2010	Japan	Authors’ diagnosis	0	2
Nakamura^29^	1994	Japan	ISGBD criteria 1992	3	5
Noel^16^	2014	France	ISGBD criteria 1990	78	37
Sumita^30^	2012	Japan	ISGBD criteria 1990	10	8

BD: Behcet’s Disease. ISGBD: International Study Group for BD. A-P-NBD: Acute parenchymal neuro BD. CP-P-NBD: Chronic progressive parenchymal neuro BD.

### Literature search strategy

In the electronic database search, we used Pubmed, EMBASE, the Cochrane Library, and Web of Science on April 1st, 2016. We used the following search formula for Pubmed without any limitation: neuro AND (Behcet’s[title] OR Behçet’s[title] OR Behcet[title] OR Behçet[title]) AND ((randomi* OR RCT OR case-control OR cohort OR cross-sectional OR epidemiol* OR prospective OR retrospective) OR ((acute OR progressive OR parenchymal OR non-parenchymal OR vasculo) AND ((symptom OR “headache” OR headache OR fever OR hemiparesis OR paraparesis OR dysarthria OR ataxia OR dementia OR psychiatr* OR seizure OR epilepsy OR incontinence OR dizziness OR vertigo OR movement OR sensory OR “cranial nerve” OR confusion OR coma OR optic OR visual OR pyramidal OR (spinal cord) OR tumor OR tumour OR (venous thrombosis)) OR (MRI OR “Magnetic resonance imaging” OR CT OR “computed tomography” OR “brain stem” OR “spinal fluid” OR CSF OR (HLA B51))))). We used similar search formulas for EMBASE, the Cochrane Library, and Web of Science (Supplementary Text 1).

We also manually searched published reviews and included original studies.

Duplicate use of the same data was excluded carefully. Two investigators (MI, NH) independently screened and scrutinized each article. Discrepancies between the investigators were resolved by discussion.

### Study selection

Two researchers (MI, NH) screened the articles for possible inclusion by reading only the title and abstract independently. Then, the two researchers independently scrutinized the full text of articles that had not been excluded by at least one researcher. Duplicate use of the same data was cautiously assessed. The final inclusion was decided by debate between the two researchers.

### Data extraction

The two researchers (MI, NH) independently extracted the data from the original studies. “First attack” was regarded as A-P-NBD. However, “second attack” and “remission and relapsing type” were excluded.

### Statistical analysis

All meta-analysis was carried out by employing the random-model generic inverse variance method using Review Manager ver. 5.3 (Cochrane Collaboration, Oxford, UK)^21^. The standard error for binary data was estimated using Wilson score interval^22^. The heterogeneity assessed by I² statistic > 75% was regarded as considerable heterogeneity^21^.

## Results

### Study search and study characteristics

**Figure 1:**
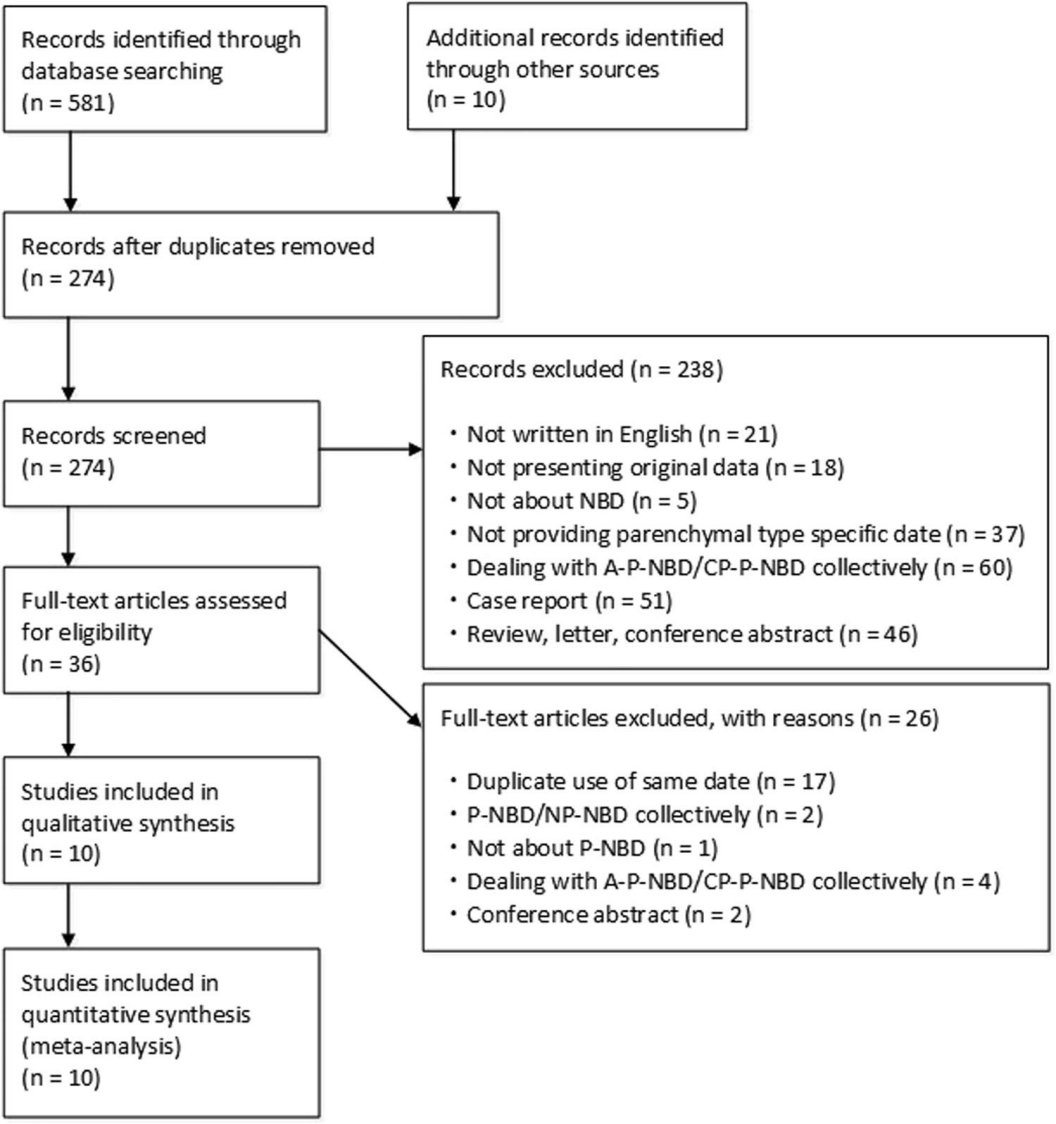
Preferred Reporting Items for Systematic Reviews and Meta- Analyses flow chart for study search.

The PRISMA flowchart for the study search is shown in Fig. 1. Of the 581 articles that we found through the primary search, 317, 238, and 26 were excluded through removal of duplication, screening, and full-article reading, respectively (Fig. 1). Notably, Hirohata *et al*. and Akman *et al*. published reports repeatedly using the same cohorts of patients, most of which were excluded from the current systematic review. Our hand search found no eligible articles.

Among the finally included 10 reports, five were from Japan, two were from Turkey, and one each was from Italy, Iran, and France^14, 16, 23–30^. The most commonly used criteria for diagnosis of BD was the International Study Group for Behçet’s disease 1990 criteria^20^, which was used in eight studies. One study used the International Study Group for Behçet’s disease 1992 criteria^31^ and the others used their own definition of BD (Table 1).

The number of participants in each study ranged from two to 115, with a median of 10. The total number of subjects was 320, consisting of 205 A-P-NBD patients and 115 CP-P-NBD patients. Of note, “second attack” and “remission and relapsing type” were excluded from this study. When acute and chronic progressive P-NBD were assessed collectively, 68.6% and 31.4% of NBD were men and women, respectively (Table 2). Onset of BD was at 39.0 years of age and that of NBD was at 42.9 years of age (Table 2).

**Table 2:** The pooled values for background characteristics, symptoms, MRI findings, and laboratories.

	Total		A-P-NBD		CP-P-NBD		p, I^2^
	N, n	Pooled (95% CI), I^2^	N, n	Pooled (95% CI), I^2^	N, n	Pooled (95% CI), I^2^	
Background characteristics							
Male (%)	7, 260	68.6 (58.4 to 78.8)%, 63%	4, 167	63.4 (49.2 to 46.9)%, 66%	7, 93	72.1 (58.1 to 42.4)%, 55%	0.39, 0%
Onset of BD (year old)	5, 254	39.0 (35.0 to 43.0)%, 83%	4, 167	39.2 (31.5 to 46.9)%, 92%	5, 87	39.3 (36.2 to 42.4)%, 34%	0.99, 0%
Onset of NBD (year old)	5, 254	42.9 (36.3 to 49.6)%, 93%	4, 167	43.1 (26.7 to 59.4)%, 96%	5, 87	43.3 (40.7 to 45.9)%, 0%	0.98, 0%
HLA-B51 positive (%)	4, 193	53.0 (35.4 to 70.6)%, 84%	2, 122	46.2 (36.7 to 55.7)%, 17%	4, 71	56.1 (24.8 to 87.4)%, 88%	0.55, 0%
Smoking (%)	1, 102	80.0 (58.4 to 100)%, 88%	1, 68	69.0 (58.2 to 79.8)%, NA	1, 34	91.0 (80.2 to 100)%, NA	0.005, 87.5%
Cyclosporin use (%)	1, 27	18.3 (0 to 48.8)%, 95%	1, 26	34.0 (23.6 to 44.4)%, NA	1, 1	2.9 (0 to 11.1)%, NA	<0.001, 95.3%
Symptom							
Fever (%)	1, 111	29.6 (0 to 82.2)%, 98%	1, 76	56.6 (45.7 to 67.5)%, NA	1, 35	2.9 (0 to 11.2)%, NA%	<0.001, 98.3%
Headache (%)	5, 248	40.7 (18.5 to 62.9)%, 94%	3, 164	45.1 (20.5 to 69.7)%, 90%	5, 84	38.9 (7.8 to 70.0)%, 92%	0.76, 0%
Cranial nerve disorder (%)	2, 123	28.3 (16.2 to 40.5)%, 46%	2, 81	27.3 (17.9 to 36.8)%, 0%	2, 42	36.6 (0 to 76.4)%, 81%	0.66, 0%
Confusion (%)	2, 133	8.5 (0.6 to 16.3)%, 35%	2, 88	4.7 (0 to 10.1)%, 0%	2, 45	17.6 (6.3 to 28.8)%, 0%	0.04, 76%
Dizziness (%)	3, 131	34.4 (2.4 to 66.4)%, 97%	2, 86	43.9 (0 to 100)%, 97%	3, 45	26.0 (0 to 53.1)%, 80%	0.62, 0%
Dementia (%)	3, 28	36.7 (9.8 to 63.7)%, 75%	2, 13	12.6 (0 to 44.3)%, 60%	3, 15	53.4 (34.4 to 72.4)%, 0%	0.03, 78.6%
Dysarthria (%)	1, 111	29.8 (6.0 to 53.6)%, 0%	1, 76	18.5 (9.8 to 27.3)%, NA	1, 35	42.9 (27.3 to 58.5)%, NA	0.008, 85.9%
Sensory disorder (%)	3, 135	20.8 (8.5 to 33.0)%, 57%	2, 88	13.5 (0 to 37.2)%, 82%	3, 47	27.3 (11.8 to 42.7)%, 26%	0.34, 0%
Cerebellar sign (%)	2, 10	81.7 (61.5 to 100)%, 0%	1, 3	66.7 (29.6 to 100)%, NA	2, 7	88.0 (63.9 to 100)%, 0%	0.34, 0%
Pyramidal sign (%)	2, 10	92.0 (72.5 to 100)%, 0%	1, 3	100.0 (66.3 to 100)%, NA	2, 7	88.0 (63.9 to 100)%, 0%	0.57, 0%
Seizure (%)	1, 115	1.7 (0 to 5.5)%, 0%	1, 78	2.6 (0 to 7.2)%, NA	1, 37	0.0 (0 to 6.5)%, NA	0.53, 0%
Hemiparesis (%)	1, 18	4.9 (0 to 19.9)%, 0%	1, 10	10.0 (0 to 31.5)%, NA	1.8	0.0 (0 to 21.0)%, NA	0.51, 0%
Sphincter disturbance (%)	2, 226	13.9 (3.5 to 24.4)%, 82%	2, 154	7.9 (0 to 19.1)%, 83%	2, 72	21.6 (12.0 to 31.3)%, 5%	0.07, 69.8%
Ataxia (%)	4, 230	38.0 (21.3 to 54.7)%, 87%	2, 154	16.5 (6.7 to 26.2)%, 63%	4, 76	53.3 (32.0 to 74.7)%, 70%	0.002, 89.5%
MRI finding							
Normal (%)	3, 135	31.5 (9.3 to 53.7)%, 91%	2, 88	34.3 (0 to 86.2)%, 95%	3, 47	28.7 (0 to 62.0)%, 85%	0.86, 0%
Brain stem any finding (%)	7, 160	62.4 (44.3 to 80.6)%, 84%	4, 102	59.7(28.9 to 90.4)%, 90%	6, 58	64.8 (38.1 to 91.4)%, 82%	0.81, 0%
Brain stem atrophy (%)	4, 32	49.1 (13.5 to 84.8)%, 87%	2, 15	11.9 (0 to 29.9)%, 0%	3, 17	76.3 (47.2 to 100)%, 65%	< 0.001, 93%
Cerebellum (%)	5, 144	26.5 (9.7 to 43.3)%, 90%	3, 93	3.5 (0 to 8.5)%, 0%	5, 51	51.3 (8.7 to 93.8)%, 93%	0.03, 79.1%
Thalamus (%)	3, 33	10.5 (0 to 22.4)%, 7%	2, 12	12.8 (0 to 44.6)%, 0%	3, 21	12.6 (0 to 27.7)%, 4%	0.99, 0%
White matter (%)	2, 29	22.3 (0 to 47.2)%, 70%	2, 21	15.9 (0 to 48.6)%, 78%	1, 8	37.5 (9.4 to 65.6)%, NA	0.33, 0%
Basal ganglia (%)	3, 127	40.5 (15.8 to 65.2)%, 90%	2, 84	48.2 (0 to 98.6)%, 94%	2, 43	35.3 (9.4 to 65.6)%, 91%	0.72, 0%
Laboratories							
CSF cell count (/mm^3^	5, 286	78.6 (48.2 to 108.9)%, 98%	4, 190	156.2 (53.8 to 258.7)%, 97%	5, 96	27.2 (0 to 54.4)%, 98%	0.02, 82.4%
CSF IL-6 (pg/mL)	4, 64	77.2 (31.8 to 122.5), 97%	2, 36	65.4 (0 to 161.3), 99%	4, 28	95.9 (21.5 to 170.2), 86%	0.62, 0%
CSF protein (mg/dL)	3, 266	89.4 (72.1 to 106.8), 99%	3, 180	112.9 (75.4 to 150.4), 99%	3, 86	78.9 (50.8 to 107.0), 99%	0.16, 50.4%

A-P-NBD: Acute parenchymal neuro Behçet’s disease CP-P-NBD: Chronic progressive parenchymal neuro Behçet’s disease.

N: number of studies. n number of patients. p: p value to compare the pooled values between A-P-NBD and CP-P-NBD.

### Comparison of A-P-NBD and CP-P-NBD

#### Background characteristics

**Figure 2:**
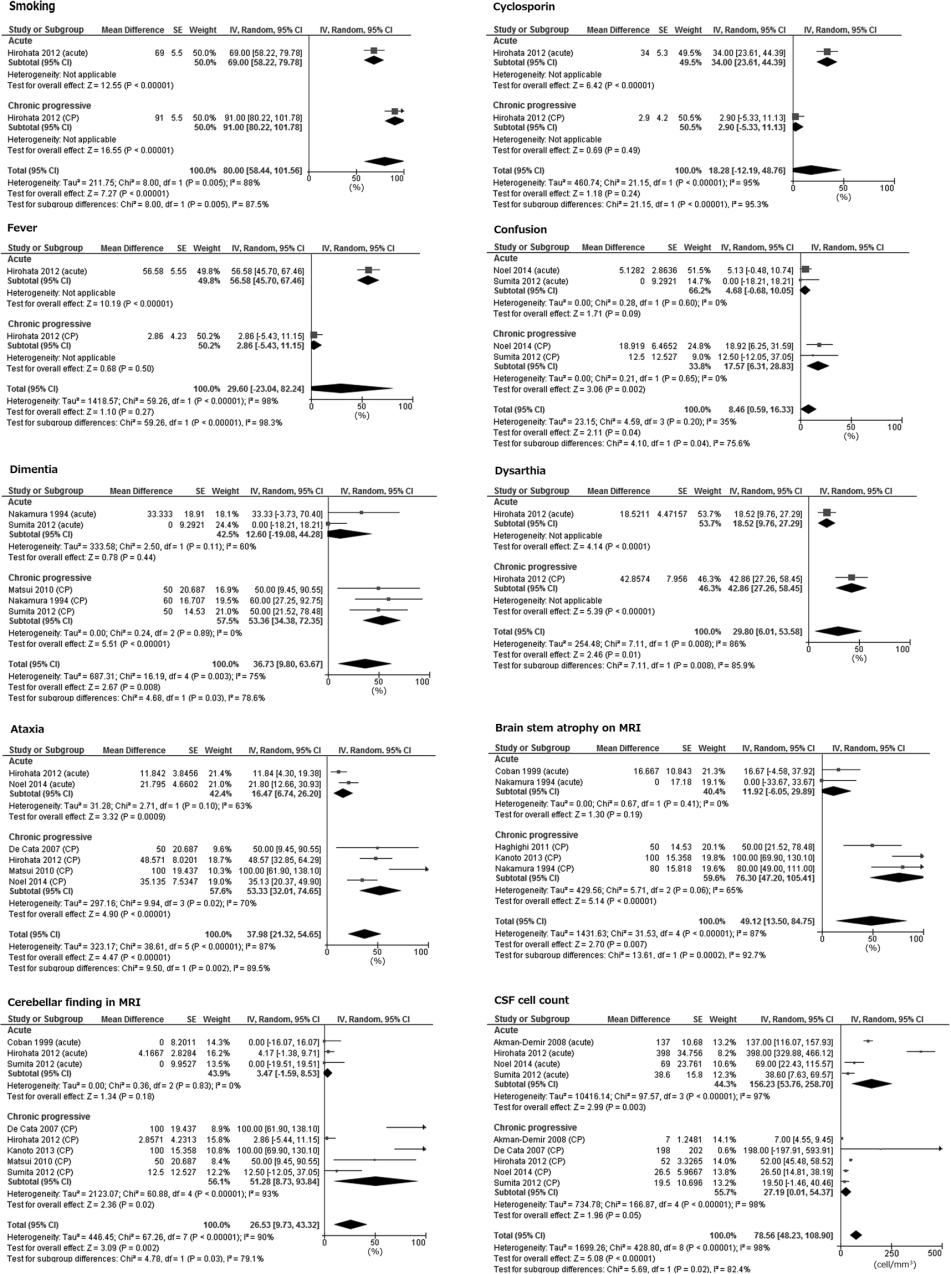
Forest plot for key findings. SE: standard error. IV: generic inverse variance method. Random: random-model meta-analysis. I^2^: I^2^ statics for heterogeneity.

No differences were found concerning age of BD onset (A-P-NBD 39.2 years, CP-P-NBD 39.3 years, p = 0.99, I^2^ = 0%) and that of NBD onset (A-P-NBD 43.1, CP-P-NBD 43.3, p = 0.98, I^2^ = 0%). Men were in the majority for both A-P-NBD and CP-P-NBD (Table 2). Our results showed no difference in the frequency of HLA-B51 positive between acute and chronic progressive P-NBD patients (A-P-NBD 46.2%, CP-P-NBD 56.1, p = 0.55, I^2^ = 0%). Although supported by only a single study, smoking history (A-P-NBD 69.0%, CP-P-NBD 91.0%, p = 0.005, I^2^ = 87.5%) and previous use of cyclosporine (A-P-NBD 34.0%, CP-P-NBD 2.9%, p < 0.001, I^2^ = 95.3%) showed distinct differences between the two groups (Table 2, Fig. 2).

### Symptoms

Fever (A-P-NBD 56.6%, CP-P-NBD 2.9%, p < 0.001, I^2^ = 98.3%) was more frequently observed in patients with A-P-NBD, whereas the frequencies of confusion (A-P-NBD 4.7%, CP-P-NBD 17.6%, p = 0.04, I^2^ = 76%), dementia (A-P-NBD 12.6%, CP-P-NBD 53.4%, p = 0.03, I^2^ = 78.6%), dysarthria (A-P-NBD 18.5%, CP-P-NBD 42.9%, p = 0.008, I^2^ = 85.9%), and ataxia (A-P-NBD 16.5%, CP-P-NBD 53.3%, p = 0.002, I^2^ = 89.5%) were higher in CP-P-NBD cases than in A-P-NBD cases (Table 2, Fig. 2). Otherwise, there were no differences in prevalence of neurological symptoms and focal signs between two groups (Table 2).

### MRI findings

Eight of 10 studies analyzed MRI findings of imaging modalities to illustrate neurological lesions in a total of 275 BD patients. Various abnormal findings were documented, though such findings were negative in 34.3% of A-P-NBD and 28.7% of CP-P-NBD (p = 0.86, I^2^ = 0%).

Brain stem atrophy on MRI (A-P-NBD 11.9%, CP-P-NBD 76.3%, p < 0.001, I^2^ = 93%) and abnormal MRI findings for cerebellum (A-P-NBD 3.5%, CP-P-NBD 51.3%, p = 0.03, I^2^ = 79.1%) were more frequently observed for patients with CP-P-NBD, though “brain stem any finding” was not different between both groups (A-P-NBD 59.7%, CP-P-NBD 64.8%, p = 0.81, I^2^ = 0%).

There were no significant differences in the prevalence of abnormal findings for thalamus (A-P-NBD 12.8%, CP-P-NBD 12.6%, p = 0.99, I^2^ = 0%), white matter (A-P-NBD 15.9%, CP-P-NBD 37.5%, p = 0.33, I^2^ = 0%), or basal ganglia (A-P-NBD 48.2%, CP-P-NBD 35.3%, p = 0.72, I^2^ = 0%).

### Laboratory data

The pooled CSF cell count of 156.2/mm^3^ in A-P-NBD was higher than that of 27.2/mm^3^ (95%CI: 0-54.4) in CP-P-NBD (p = 0.02). On the other hand, CSF IL-6 levels (A-P-NBD 65.4 pg/mL, CP-P-NBD 95.9 pg/mL, p = 0.62, I^2^ = 0%) and CSF protein levels (A-P-NBD 112.9 mg/dL, CP-P-NBD 78.9 mg/dL, p = 0.16, I^2^ = 50.4%) were not significantly different between the two groups.

## Discussion

In this systematic review, we analyzed the characteristics in 320 patients with P-NBD. To the best of our knowledge, our report is the first systematic review to clarify the differences between A-P-NBD and CP-P-NBD. We showed that the clinical features in the A-P-NBD and CP-P-NBD were distinct besides disease duration and chronological clinical course. The patients with A-P-NBD generally present episodic meningitis and/or brainstem encephalitis with high fever and elevated CSF cell count. On the other hand, confusion, dementia, dysarthria, and ataxia were more common in CP-P-NBD. Besides the gradual progression of these symptoms, cerebellar and brain stem atrophy shown by MRI appear shared features with neurodegenerative disorders rather than other immune mediated neurological diseases. These features in individual clinical phenotypes are incorporated in preliminary diagnostic criteria of A-P-NBD and CP-P-NBD proposed by Hirohata and the Behcet’s Disease Research Committee, Ministry of Health, Welfare, and Labour, Japan^14^.

It is important that physicians are aware of these differences of clinical presentation between the two types of P-NBD, because the distinction may help with decision-making on treatment and expecting prognosis. Currently, no treatment option for P-NBD have been supported by randomized trials^9, 32, 33^. Besides low incidence of P-NBD, the clinical heterogeneity among P-NBD cases makes it difficult to obtain evidence for the therapeutic strategy. The current study revealed clearly distinctions in clinical symptoms, MRI findings, and CSF data between A-P-NBD and CP-P-NBD, suggesting differences in pathophysiology between them. It is essential to differentiate the two types of P-NBD to determine the best therapeutic strategy. Indeed, clinical manifestations of A-P-NBD subside in response to moderate to high dose of corticosteroid and the relapse is significantly suppressed by colchicine^34^. On the other hand, few studies have shown therapeutic effects of corticosteroid and conventional immunosuppressants on CP-P-NBD except methotrexate^35^. Methotrexate is recommended as the first line therapy for CP-P-NBD in the guidelines for management of NBD from Behcet’s Disease Research Committee, Ministry of Health, Welfare, and Labour, Japan. Favorable clinical outcomes of TNF inhibitors for both types of NBD have been accumulated including prospective study^36^.

Cyclosporine neurotoxicity in BD patients is well recognized as mentioned in EULAR recommendations for the management of BD^32^. The previous use of cyclosporine was exclusively associated with A-P-NBD, though it was analyzed in only one study^14^. The drug related A-P-NBD is generally reversible by discontinuation of cyclosporine with corticosteroids, as the natural onset disease.

Distinction between A-P-NBD and CP-P-NBD was originally advocated in Japan^17, 18^. Ideguchi *et al*.^13^ showed the difference of symptoms and MRI findings between the two categories of P-NBD by reviewing 38 patients with A-P-NBD and 15 patients with CP-P-NBD. They revealed that fever and headache were prevalent for acute NBD and that personality change, sphincter disturbance, involuntary movement, and ataxia were predominant in patients with CP-P-NBD. These findings were generally compatible with our analysis.

Hirohata *et al*.^14^ showed that the CSF cell count is increased in the acute phase of A-P-NBD. CSF IL-6 level was higher than normal range in both A-P-NBD and CP-P-NBD groups (Table 2). CSF IL-6 level is often elevated in meningitis patients, thus, it is reasonable that CSF IL-6 level is increased at acute phase and decreased along the remission^14^. On the other hand, persistent elevation of CSF IL-6 is characteristic for CP-P-NBD (Fig. 2)^37^. Akman-Demir *et al*.^23^. in Turkey also showed similar CSF findings in A-P-NBD and CP-P-NBD, but not NP-NBD^23^.Furthermore, Kikuchi *et al*. showed that the progression rate of brain stem atrophy evaluated by MRI was closely correlated with CSF IL-6 elevation^38^. Interestingly, CSF IL-6 could be an activity marker for P-NBD patients even for those having normal range of CSF cell count and protein level^23^. This is not the case for serum IL-6 level^23, 39^. A few case reports have shown therapeutic effect of anti-IL-6 receptor antibody, tocilizumab, on CP-P-NBD^40, 41^, though this should be validated in a larger number of patients.

Both genetic and environmental factors have been considered to affect onset and clinical course of BD^42^. Especially, a number of studies have focused on association of HLA-B51 with clinical phenotypes of BD. A meta-analysis by Maldini *et al*. have shown that frequency of HLA–B51 is not higher in a whole of NBD patients than other subtypes of BD patients^43^. The largest study in the included researches was carried out by Noel *et al*. in France (Table 2), whose goal was to reveal the prognostic factor of P-NBD^16^. Likewise, Aramaki *et al*. also identified HLA-B51 and smoking as independent predisposing factors to CP-P-NBD^44^. These findings suggested that HLA-B51 is associated with unfavorable clinical course of NBD. However, the present study failed to show difference in the prevalence of HLA-B51 between A-P-NBD and CP-P-NBD patients (Table 2).

We need to comment on some limitations of the current study. First, NBD diagnosis was not oriented from the latest recommendation^7^. Similarly, there might be a slight inconsistency of definition of A-P-NBD and CP-P-NBD between studies. Therefore, diagnosis of NBD and nomenclatures of the clinical subtypes were not necessarily consistent among the studies, though the concepts were similar. In addition, clinical symptoms and MRI findings were evaluated by each investigator’s criteria. Second, as many as five out of the 10 included studies were from Japan. However, the data sources in this study are widely distributed in both sides of the Silk Road; 139, 115, 52, 8, and 2 cases were derived from reports from Japan, France, Turkey Iran, and Italy, minimizing regional bias. Third, we could not directly access the raw data of each patient; thus, detailed analysis concerning combinations and chronological change of signs and symptoms were not plausible. Further analysis using individual patient data may be interesting.

## Conclusion

In conclusion, we performed a first systematic review and meta-analysis to discriminate the clinical presentation of A-P-NBD and CP-P-NBD. According to 10 studies with 205 A-P-NBD cases and 111 CP-P-NBD cases, fever and elevated CSF cell count featured A-P-NBD, whereas CP-P-NBD was characterized by sphincter disturbances, ataxia, dementia, confusions, brain stem atrophy, and abnormal MRI findings in cerebellum. Thus, it is important to recognize A-P-NBD and CP-P-NBD separately for management of NBD patients.

## Electronic supplementary material

Supplementary information
