# Supplementary material for: Distinct clinical features between acute and chronic progressive parenchymal neuro-Behçet disease: meta-analysis
Source: Sci Rep. 2017 Aug 31;7:10196. doi: 10.1038/s41598-017-09938-z (PMC5579041; doi:10.1038/s41598-017-09938-z)
Supplement: Supplementary file 1 — Supplementary information [file 41598_2017_9938_MOESM1_ESM.doc]

**Distinct clinical features between acute and chronic progressive parenchymal**

**neuro-Behçet disease: meta-analysis**

**<<Supplementary File>>**

Authors

^1)^Mizuho Ishido, ^2)^Nobuyuki Horita, ^1)^Masaki Takeuchi, ^1)^Etsuko Shibuya,

^1)^Takahiro Yamane, ^1)^Tatsukata Kawagoe, ^1)^Takehito Ishido, ^3)^Kaoru Minegishi, ^3)^Ryusuke Yoshimi, ^3)^Yohei Kirino, ^4)^Shunsei Hirohata, ^5)^Yoshiaki Ishigatsubo,

^6)^Mitsuhiro Takeno, ^2)^Takeshi Kaneko, ^1)^Nobuhisa Mizuki.

**Supplementary Text 1. Search formulas**

*Web of Science Core Collection*

Advanced search without limitation

#1 TS=(neuro)

#2 TI=(Behcet's OR Behçet's OR Behcet OR Behçet)

#3 TS=(randomi* OR RCT OR case-control OR cohort OR cross-sectional OR epidemiol* OR prospective OR retrospective)

#4 TS=(acute OR "chronic progressive" OR parenchymal OR non-parenchymal)

#5 TS=((symptom OR "head ache" OR headache OR fever OR hemiparesis OR paraparesis OR dysarthria OR ataxia OR dementia OR psychiatr* OR seizure OR epilepsy OR incontinence OR dizziness OR vertigo OR sensory OR "cranial nerve" OR confusion OR coma OR optic OR visual OR pyramidal) OR (MRI OR "Magnetic resonance imaging" OR CT OR "computed tomography" OR "brain stem" OR "spinal fluid" OR CSF OR (HLA B51)))

#6: #4 AND #5

#7: #3 OR #6

#8: #1 AND #2

#9: #7 AND #8

*Cochrane Library hosts the Central Register of Controlled Trials*

Title/Abstract/Keywords search without limitation

(neuro-Behcet's OR neuro-Behçet's OR neuro-Behcet OR neuro-Behçet)

*Embase*

Without limitation

neuro AND (behçet*:ti OR behcet*:ti) AND (randomi* OR rct OR 'case control' OR cohort OR 'cross sectional' OR epidemiol* OR prospective OR retrospective OR (acute OR progressive OR parenchymal OR 'non parenchymal' OR vasculo AND ('symptom'/exp OR symptom OR 'head ache'/exp OR 'head ache' OR 'headache'/exp OR headache OR 'fever'/exp OR fever OR 'hemiparesis'/exp OR hemiparesis OR 'paraparesis'/exp OR paraparesis OR 'dysarthria'/exp OR dysarthria OR 'ataxia'/exp OR ataxia OR 'dementia'/exp OR dementia OR psychiatr* OR 'seizure'/exp OR seizure OR 'epilepsy'/exp OR epilepsy OR 'incontinence'/exp OR incontinence OR 'dizziness'/exp OR dizziness OR 'vertigo'/exp OR vertigo OR 'movement'/exp OR movement OR sensory OR 'cranial nerve'/exp OR 'cranial nerve' OR 'confusion'/exp OR confusion OR 'coma'/exp OR coma OR optic OR visual OR pyramidal OR (spinal AND cord) OR 'tumor'/exp OR tumor OR 'tumour'/exp OR tumour OR (venous AND ('thrombosis'/exp OR thrombosis)) OR 'mri'/exp OR mri OR 'magnetic resonance imaging'/exp OR 'magnetic resonance imaging' OR ct OR 'computed tomography'/exp OR 'computed tomography' OR 'brain stem'/exp OR 'brain stem' OR 'spinal fluid'/exp OR 'spinal fluid' OR 'csf'/exp OR csf OR ('hla'/exp OR hla AND b51))))
